# *MMP-8* C-799 T, Lys460Thr, and Lys87Glu variants are not related to risk of cancer

**DOI:** 10.1186/s12881-019-0890-z

**Published:** 2019-10-21

**Authors:** Li-Feng Zhang, Li-Jie Zhu, Wei Zhang, Wei Yuan, Ning-Hong Song, Li Zuo, Yuan-Yuan Mi, Zeng-Jun Wang, Wei Zhang

**Affiliations:** 1 Department of Urology, The First Affiliated Hospital of Nanjing Medical University, Nanjing, China; 2 Department of Urology, The Affiliated Changzhou No.2 People’s Hospital of Nanjing Medical University, Changzhou, 213003 Jiangsu Province China; 3 Department of Urology, Affiliated Hospital of Jiangnan University, Wuxi, 214000 China; 4 Department of Oncology, Taizhou People’s Hospital, Taizhou, 225300 Jiangsu China; 5 Department of Cardiology, Taizhou People’s Hospital, Taizhou, 225300 Jiangsu China

**Keywords:** *MMP*-8, Variant, Cancer, Analysis

## Abstract

**Background:**

Several studies have focused on the relationship between *MMP-8* variants and cancer risk, but they have been unsuccessful in drawing reliable conclusions.

**Methods:**

We employed odds ratio (OR) together with 95% confidence interval (CI) to assess the correlation between *MMP-8* C-799 T, Lys460Thr, and Lys87Glu polymorphisms and cancer risk. We further employed in silico tools to evaluate the effect of *MMP-8* expression on cancer susceptibility and overall survival time.

**Results:**

A total of 8140 patients with malignant carcinoma and 10,529 healthy individuals (control) were enrolled. Overall, the analysis showed that the relationship between three *MMP-8* variants and cancer susceptibility was not significant (allelic contrast, C-799 T: OR = 0.98, 95% CI = 0.92–1.04, *P*_heterogeneity_ = 0.068; Lys460Thr: OR = 0.94, 95% CI = 0.67–1.32, *P*_heterogeneity_ = 0.905; Lys87Glu: OR = 1.05, 95% CI = 0.93–1.18, *P*_heterogeneity_ = 0.968). Similar results were observed in subgroup analysis by ethnicity, cancer type, and source of control. In silico analysis indicated that *MMP-8* expression was elevated in bladder cancer tissue compared to that in the control. However, both the higher and lower *MMP-8* expression groups did not show an impact on the overall survival time of the patients.

**Conclusions:**

*MMP-8* C-799 T, Lys460Thr, and Lys87Glu variants are not participant with the susceptibility of cancer.

## Background

It is well-known that the development of carcinoma is complex and has not been completely clarified. Hereditary material and genetic polymorphisms may probably have an impact on cancer susceptibility and play a crucial role in the tumorigenesis of numerous carcinomas [1–4]. Previous studies have shown evidence that the genetic aspects measured by single nucleotide polymorphisms (SNPs) might be associated with cancer susceptibility [5–8]. Matrix metalloproteinases (MMPs) belong to a family of endopeptidases that can degrade various extracellular matrix proteins and treat numerous extracellular matrix (ECM) components [9, 10]. Accumulated evidence has indicated that MMPs may have a critical role in cell inflammation, migration and carcinogenesis [11–13]. Increased levels of MMPs have been observed in the specimens of a number of cancer subjects, such as urinary bladder cancer, lung cancer, breast cancer, and malignant melanoma [14]. Among the MMPs, MMP8 is a collagen-cleaving enzyme present in connective tissue. The MMP8 is not only produced by neutrophils but also synthesized by a series of malignant tumor cells [15, 16]. High level of MMP8 was reported in the fluid of ovarian cancer compared to control tissue [17].

Previous studies have showed evidence that genetic mutations and variants can predispose for malignant tumors [18, 19]. Human *MMP-8* gene comprises twelve exons and is located on chromosome 11q22.3 [20]. Polymorphisms of *MMP8* can lead to gene dysfunction, microenvironment disorder and potential carcinogenesis. It has been reported that several single nucleotide polymorphisms (SNPs) of *MMP8* can influence the gene expression by altering its promoter activity. In addition, the T allele of *MMP8* C-799 T variant was reported to be related to breast carcinoma susceptibility and lymph node metastasis among Asian and Caucasian population [21]. Furthermore, electrophoresis mobility shift assays have demonstrated that the difference in nucleoprotein binding to oligodeoxynucleotides was correlated with *MMP8* C-799 T variation [22]. However, Wieczorek et al. indicated that genetic variation in *MMP8* C-799 T was not associated with urinary bladder cancer susceptibility in Caucasian descendants [23].

A number of studies have evaluated the association of *MMP8* genetic variants including C-799 T (rs11225395 C/T), Lys460Thr (rs35866072 A/C), and Lys87Glu (rs1940475 G/A) SNPs, with cancer risk. Research on these *MMP8* variations have been carried out in numerous countries, such as USA, Mexico, Poland, India, Korea, and China [23–35]. Nevertheless, there were controversial results on the relationship between *MMP8* variations and various cancers among different case control studies. Therefore, we conducted a comprehensive analysis based on accumulated data of all eligible studies to investigate the impact of *MMP-8* C-799 T, Lys460Thr, and Lys87Glu polymorphisms on overall cancer susceptibility.

## Methods

### Database searching and screening process

Comprehensive literature search on PubMed, Web of Science, EMBASE (Excerpta Medica Database), and SinoMed (China Wanfang Databases) was carried out to identify all eligible case-control studies, prior to June 2019. Valid keyword search strings are as follows: (MMP-8 OR matrix metalloproteinases 8) AND (polymorphism OR variant OR mutation OR SNP) AND (carcinoma OR tumor OR malignancy OR cancer). Furthermore, we independently retrieved the references in the identified articles to screen other available studies, with no language restriction. For studies with overlapping data, we selected the most recently published ones.

### Inclusion and exclusion criteria

Eligible studies should meet the following inclusion criteria: (a) evaluate the relationship between *MMP-8* C-799 T, Lys460Thr, and Lys87Glu polymorphisms and cancer risk; (b) case-control study; and (c) contain available data on the frequency of genotypes. We independently excluded unpublished case reports, letters, reviews, meta-analyses, or missing genotype data for C-799 T, Lys460Thr, and Lys87Glu variants. We also excluded the studies that focused only on the case population.

### Data extraction and quality assessment

Two authors (LFZ and YYM) independently completed the data extraction based on the selection criteria. Any potential disagreement was discussed comprehensively to obtain a final consensus. The main features of included studies are summarized, which includes: first author’s name, year of publication, origin and race, type of cancer, age range, total number of participants, genotyping assay of *MMP-8* C-799 T, Lys460Thr, and Lys87Glu variants in cases and controls, *P*-value of Hardy-Weinberg equilibrium (HWE) in controls. The quality score of the eligible studies was evaluated by Newcastle-Ottawa Scale (NOS). The research was regarded as high-quality if it acquired six or more stars.

### Statistical analysis

We calculated the OR with 95% CI to investigate the strength of the relationship between *MMP-8* C-799 T, Lys460Thr, and Lys87Glu polymorphisms and cancer susceptibility. A total of five genetic models were adopted: allelic contrast (M-allele vs. W-allele, for C-799 T, T vs. C; for Lys460Thr, C vs. A; for Lys87Glu, A vs. G), homozygote model (MM vs. WW, for C-799 T, TT vs. CC; for Lys460Thr, CC vs. AA; for Lys87Glu, AA vs. GG), heterozygote model (MW vs. WW, for C-799 T, TC vs. CC; for Lys460Thr, CA vs. AA; for Lys87Glu, AG vs. GG), dominant comparison (MM + MW vs. WW, for C-799 T, TT + TC vs. CC; for Lys460Thr, CC+ CA vs. AA; for Lys87Glu, AA + AG vs. GG), recessive comparison (MM vs. MW + WW, for C-799 T, TT vs. TC + CC; for Lys460Thr, CC vs. CA + AA; for Lys87Glu, AA vs. AG + GG). *Q*-test was utilized to estimate the heterogeneity among enrolled researches. If the heterogeneity was absent (*P* > 0.05), the fixed-effects model was employed [36]; alternatively, the random-effects model was performed [37]. Stratified analyses were carried out according to race (Asian, Caucasian, and Latin), type of cancer (bladder cancer and other cancers), and source of control. Hardy-Weinberg equilibrium (HWE) in the control group was calculated using a Chi-squared test. Begg’s funnel plot and the Egger’s test were both performed to measure the possible publication bias. *P* values of Begg’s and Egger’s test more than 0.05 indicated the absence of publication bias. The STATA software (Version 11.0, Stata Corporation, College Station, TX, USA) was adopted for all the above analyses.

### In silico analysis of MMP-8

To further investigate whether the expression of *MMP-8* has an impact on tumorigenesis, we employed the online TCGA database to evaluate the *MMP-8* expression in bladder cancer tissue and control counterparts. The effect of the expression of *MMP-8* on bladder cancer patients’ overall survival time was also assessed. Furthermore, we adopted bioinformatics tools, like Polyphen-2 (http://genetics.bwh.harvard.edu/pph2/), to predict the role of *MMP-8* SNPs at the protein level.

## Results

### Characteristics of included studies

**Fig. 1:**
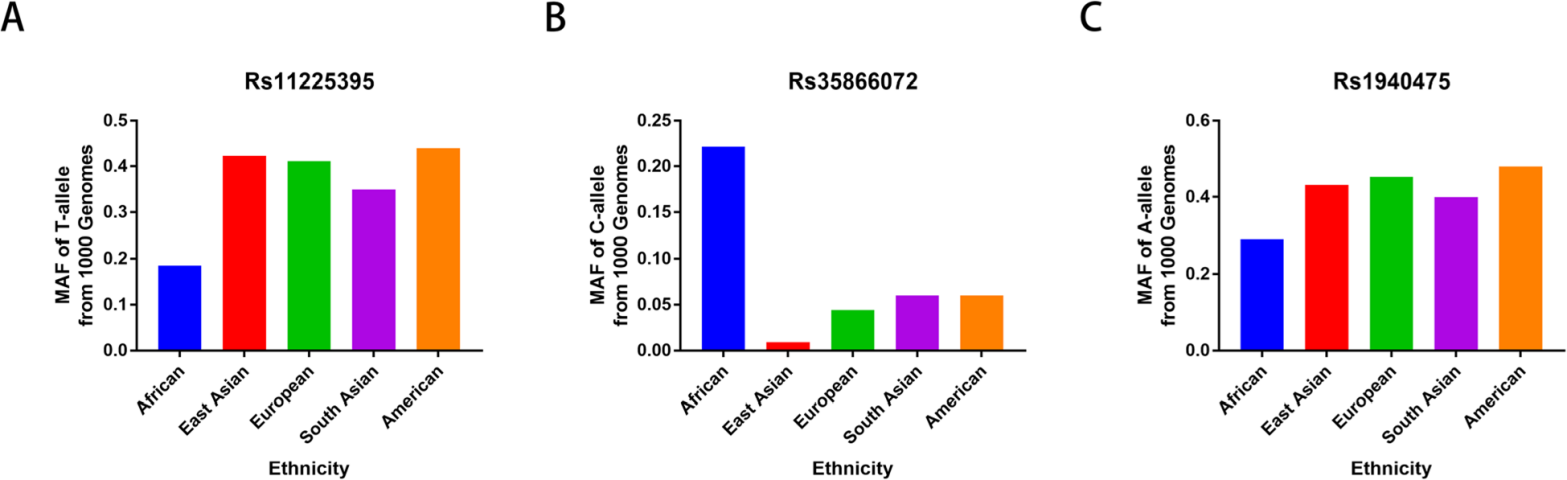
Minor allele and major allele frequencies for *MMP-8* C-799 T (**a**), Lys460Thr (**b**), and Lys87Glu (**c**) variants in controls stratified by ethnicity. Vertical line, allele frequency; Horizontal line, allele type

Main characteristics of the eligible articles as well as the genotyping assay results of *MMP-8* C-799 T, Lys460Thr, and Lys87Glu variants have been summarized in Table 1. A total of 13 publications containing 19 case-control studies for the *MMP-8* polymorphisms in compliance with the inclusion criteria were finally identified in the present analysis. All eligible studies had NOS score more than 6. There were 4372 cancer patients and 5066 control participants for the analysis of *MMP-8* C-799 T variant. Five articles were acquired for assessing the association of *MMP-8* Lys460Thr variant on cancer susceptibility, including 3019 cases and 3544 control subjects. There were 749 cases and 1919 controls on the Lys87Glu polymorphism. The MAFs (minor allele frequencies) of *MMP-8* C-799 T variants were shown in Fig. 1: African, 0.185; East Asian, 0.423; European, 0.411; South Asian, 0.350; and American, 0.440. For *MMP-8* Lys460Thr polymorphism: African, 0.222; East Asian, 0.009; European, 0.044; South Asian, 0.060; and American, 0.060. The MAFs for Lys87Glu variant were: African, 0.290; East Asian, 0.432; European, 0.453; South Asian, 0.400; and American, 0.480. In stratified analysis by race, 13 case-control studies were conducted on Asian descendants; five were based on the Caucasian population and one was based on Latin descendants. In stratified analysis by cancer type, four studies concerned bladder cancer. The rest were focused on other cancers, such as lung cancer, hepatocellular carcinoma, malignant melanoma, oral cancer, ovary cancer, and gastric cancer. In stratified analysis by the source of control, 12 studies were population-based controls, and the rest seven were hospital-based studies.

**Table 1:** Basic information of included studies for *MMP*-8 C-799 T, Lys460Thr, and Lys87Glu variants and overall cancer risk

Author	Year	Origin	Cancer Type	Race	Source	Case	Control	Case			Control			*P*_HWE_	Method	Age range		NOS
C-799 T(rs11225395)								TT	TC	CC	TT	TC	CC			Case	Control	
Tsai	2018	Taiwan	Bladder cancer	Asian	PB	375	375	37	152	186	38	140	197	0.082	PCR-RFLP	mean 61.4	mean 62.9	7
Hsiao	2018	Taiwan	Breast cancer	Asian	PB	1232	1232	118	466	648	131	468	633	0.002	PCR	NA	NA	7
Pei	2017	Taiwan	Leukemia	Asian	PB	266	266	29	98	139	32	105	129	0.145	PCR-RFLP	mean 7.0	mean 8.3	7
Shen	2017	Taiwan	Lung cancer	Asian	PB	358	716	40	130	188	92	273	351	0.001	PCR-RFLP	mean 64.0	mean 64.8	7
Hung	2017	Taiwan	Oral cancer	Asian	PB	788	956	90	284	414	126	364	466	< 0.001	PCR	mean 55.8	mean 56.6	7
Arechavaleta	2014	Mexico	Ovary cancer	Latin	HB	51	37	12	24	15	5	26	6	0.013	PCR-RFLP	49 (25–82)	39 (13–77)	7
Wieczorek	2013	Poland	Bladder cancer	Caucasian	HB	241	199	44	125	72	38	101	60	0.697	RT PCR	66.3 ± 10.6	66.1 ± 10.4	8
Srivastava	2013	India	Bladder cancer	Asian	HB	200	200	11	90	99	24	84	92	0.478	PCR-based	58.5 ± 12.4	56.8 ± 10.8	8
Kim	2011	Korea	Gastric cancer	Asian	HB	148	315	14	67	67	38	127	150	0.172	GoldenGate	mean 57.8	mean 55.2	7
Debniak	2011	Poland	MM	Caucasian	PB	296	290	58	152	86	43	134	113	0.750	TaqMan	mean 56.0	mean 55.0	8
Qiu	2008	China Mainland	HCa	Asian	HB	417	480	81	196	140	80	216	184	0.223	PCR-RFLP	NA	NA	8
Lys460Thr								CC	CA	AA	CC	CA	AA					
Tsai	2018	Taiwan	Bladder cancer	Asian	PB	375	375	3	7	365	4	9	362	< 0.001	PCR-RFLP	mean 61.4	mean 62.9	7
Hsiao	2018	Taiwan	Breast cancer	Asian	PB	1232	1232	7	26	1199	8	23	1201	< 0.001	PCR	NA	NA	7
Pei	2017	Taiwan	Leukemia	Asian	PB	266	266	0	2	264	0	3	263	0.926	PCR-RFLP	mean 7.0	mean 8.3	7
Shen	2017	Taiwan	Lung cancer	Asian	PB	358	715	0	3	355	0	4	711	0.940	PCR-RFLP	mean 64.0	mean 64.8	7
Hung	2017	Taiwan	Oral cancer	Asian	PB	788	956	0	7	781	0	10	946	0.871	PCR	mean 55.8	mean 56.6	7
Lys87Glu								AA	AG	GG	AA	AG	GG					
Nan	2008	USA	Skin Cancer	Caucasian	PB	206	827	56	104	46	222	409	196	0.776	TaqMan	mean 63.4	mean 64.5	7
Kader	2006	USA	Invasive BCa	Caucasian	HB	236	546	61	106	69	115	278	153	0.587	RT PCR	65 (21–88)	64 (24–89)	7
Kader	2006	USA	Superficial BCa	Caucasian	HB	307	546	70	152	85	115	278	153	0.587	RT PCR	65 (21–88)	64 (24–89)	7

*HB* hospital-based, *HCa* Hepatocellular carcinoma, *BCa* bladder cancer, *MM* Malignant melanoma, *PB* population-based, *RT* real time, *PCR-RFLP* polymerase chain reaction and restrictive fragment length polymorphism, *NA* not available, *HWE* Hardy-Weinberg equilibrium of controls

### Quantitative synthesis

**Fig. 2:**
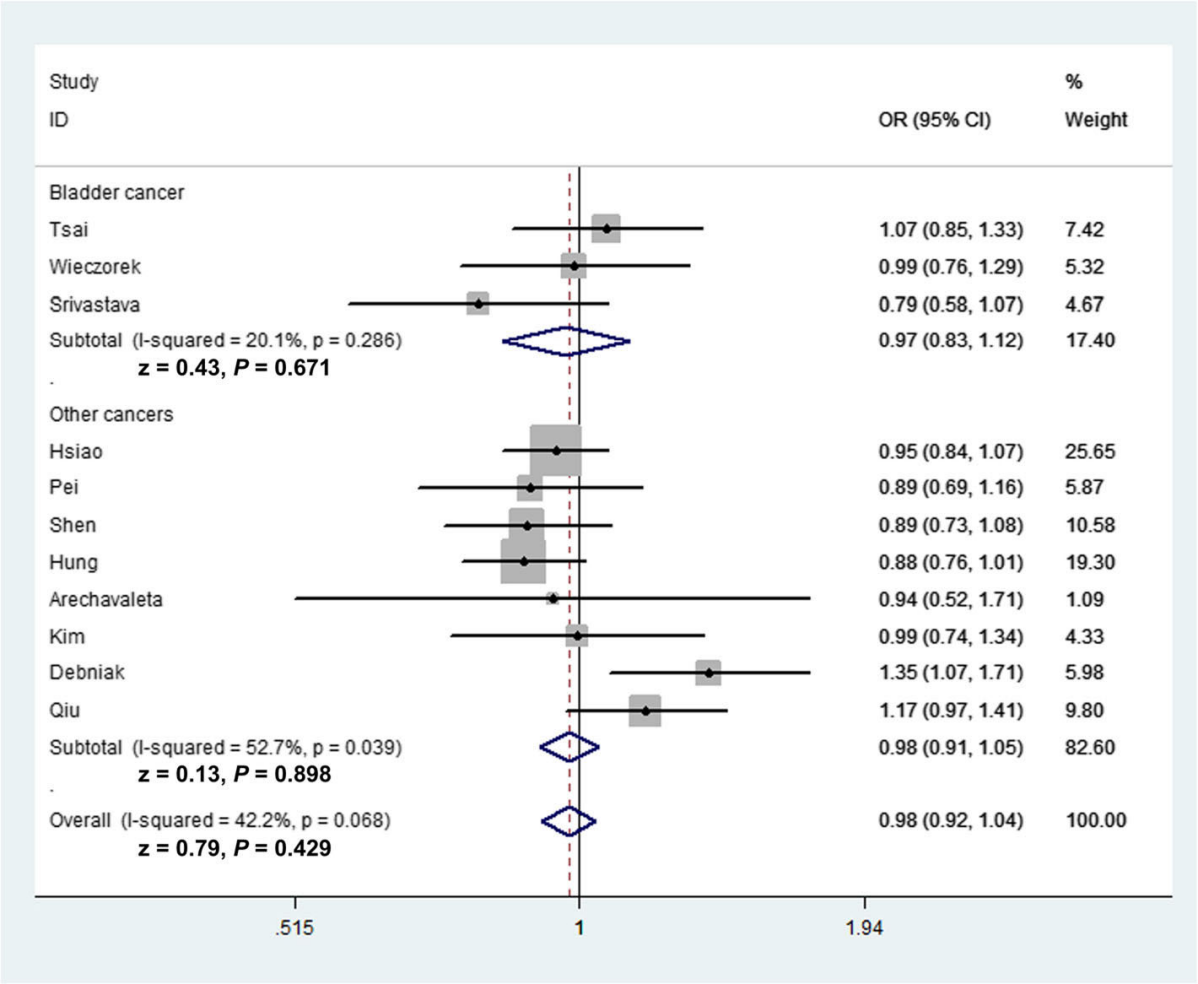
Forest plot of allelic contrast of *MMP-8* C-799 T polymorphism in the stratified analyses by cancer type (fixed-effects)

**Fig. 3:**
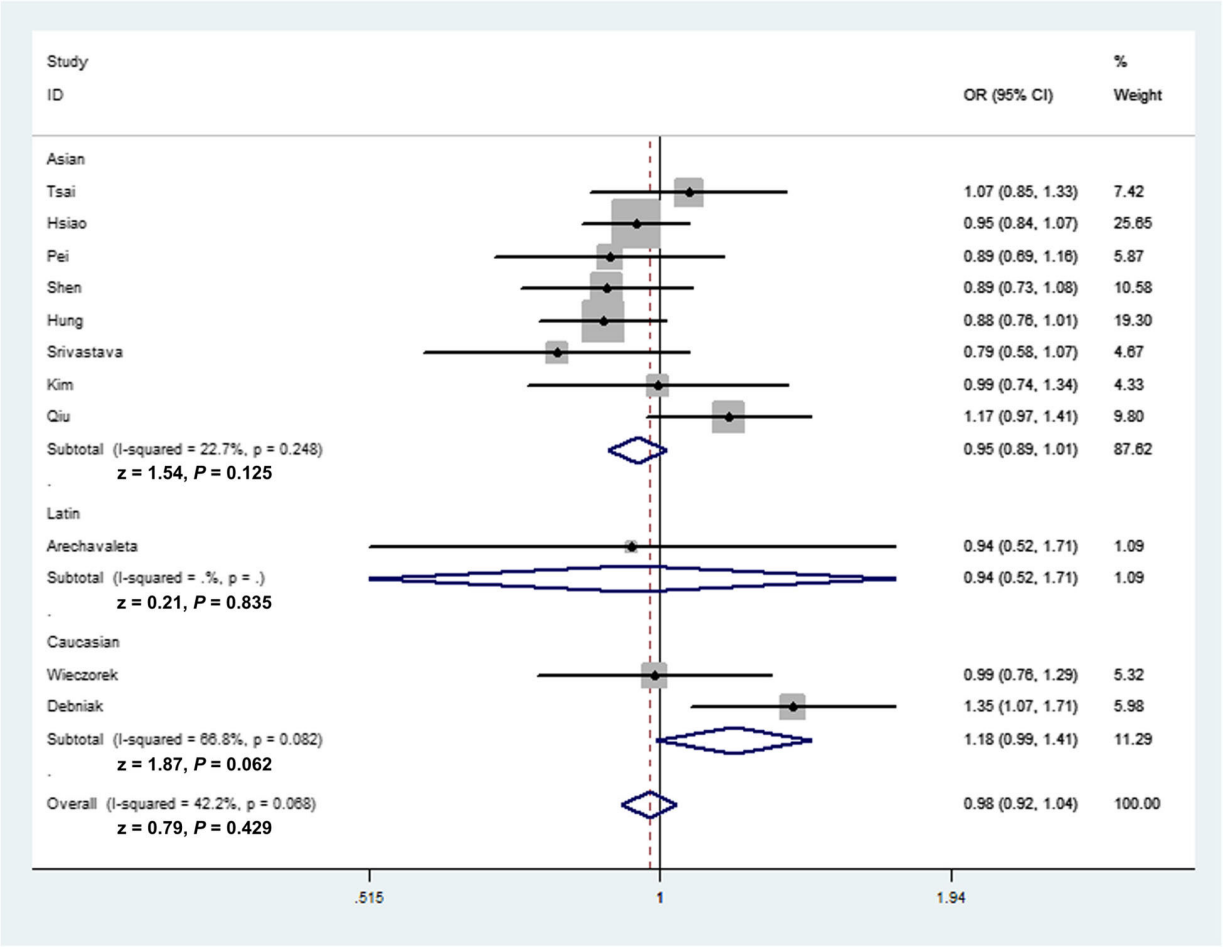
Forest plot of cancer risk associated with *MMP-8* C-799 T variant (allelic comparison of T-allele vs. C-allele, fixed-effects) in the stratified analyses by ethnicity

Summarized results and details of the present analyses for the three *MMP-8* polymorphisms and cancer risk are provided in Table 2. Overall analysis indicated that the relationship between the three *MMP-8* variants and cancer susceptibility was not significant. The *MMP-8* C-799 T variant is not associated with the susceptibility of cancer under all genetic models (allele contrast: OR = 0.98, 95% CI = 0.92–1.04, *P*_heterogeneity_ = 0.068, *P* = 0.429; TT vs. CC: OR = 0.94, 95% CI = 0.82–1.07, *P*_heterogeneity_ = 0.097, *P* = 0.362; heterozygote comparison: OR = 1.00, 95% CI = 0.92–1.09, *P*_heterogeneity_ = 0.193, *P* = 0.992; TT + TC vs. CC: OR = 0.98, 95% CI = 0.90–1.07, *P*_heterogeneity_ = 0.086, *P* = 0.666; recessive model: OR = 0.94, 95% CI = 0.83–1.07, *P*_heterogeneity_ = 0.249, *P* = 0.348). In subgroup analysis by cancer type, we also indicated no relationship between *MMP-8* C-799 T variant and bladder cancer (T vs. C: OR = 0.97, 95% CI = 0.83–1.12, *P* = 0.671, Fig. 2; TT vs. CC: OR = 0.85, 95% CI = 0.61–1.17, *P* = 0.316; TC vs. CC: OR = 1.08, 95% CI = 0.87–1.33, *P* = 0.492; TT + TC vs. CC: OR = 1.02, 95% CI = 0.84–1.25, *P* = 0.813; TT vs. TC + CC: OR = 0.83, 95% CI = 0.61–1.12, *P* = 0.219) or other cancers (T vs. C: OR = 0.99, 95% CI = 0.89–1.11, *P* = 0.898; TT vs. CC: OR = 0.96, 95% CI = 0.83–1.11, *P* = 0.578; TC vs. CC: OR = 0.99, 95% CI = 0.89–1.09, *P* = 0.763; TT + TC vs. CC: OR = 1.00, 95% CI = 0.86–1.16, *P* = 0.965; TT vs. TC + CC: OR = 0.97, 95% CI = 0.84–1.11, *P* = 0.631). In stratified analysis by race and source of control, no significant association between this polymorphism and cancer susceptibility was demonstrated (Fig. 3). For the *MMP-8* Lys460Thr variant, we also indicated no major association of this variant on cancer risk (C vs. A: OR = 0.94, 95% CI = 0.67–1.32, *P*_heterogeneity_ = 0.905, *P* = 0.729; CC vs. AA: OR = 0.83, 95% CI = 0.36–1.93, *P*_heterogeneity_ = 0.859, *P* = 0.669; CA vs. AA: OR = 1.00, 95% CI = 0.66–1.50, *P*_heterogeneity_ = 0.904, *P* = 0.994; TT + TC vs. CC: OR = 0.96, 95% CI = 0.67–1.40, *P*_heterogeneity_ = 0.886, *P* = 0.848; recessive model: OR = 0.83, 95% CI = 0.36–1.93, *P*_heterogeneity_ = 0.866, *P* = 0.669, Table 2). In addition, similar results were revealed for the association between the *MMP-8* Lys87Glu variant and cancer risk in allelic contrast (OR = 1.05, 95% CI = 0.93–1.18, *P* value for heterogeneity = 0.968, *P* = 0.430); homozygote model (OR = 1.11, 95% CI = 0.88–1.42, *P*_heterogeneity_ = 0.953, *P* = 0.377); heterozygote comparison (OR = 0.96, 95% CI = 0.78–1.18, *P* value for heterogeneity = 0.647, *P* = 0.722); dominant model (OR = 1.01, 95% CI = 0.83–1.22, *P*_heterogeneity_ = 0.863, *P* = 0.928), and recessive comparison (OR = 1.13, 95% CI = 0.93–1.38, *P*_heterogeneity_ = 0.604, *P* = 0.222).

**Table 2:** Stratified analyses of *MMP*-8 C-799 T, Lys460Thr, and Lys87Glu variants on overall cancer risk

Variables	*N*	Case/	OR(95% CI) *P*h *P*	OR(95% CI) *P*h *P*	OR(95% CI) *P*h *P*	OR(95% CI) *P*h *P*	OR(95% CI) *P*h *P*
		Control	M-allele vs. W-allele	MM vs. WW	MW vs. WW	MM + MW vs. WW	MM vs. MW + WW
C-799 T							
Total	11	4372/5066	0.98 (0.92–1.04) 0.068 0.429	0.94 (0.82–1.07) 0.097 0.362	1.00 (0.92–1.09) 0.193 0.992	0.98 (0.90–1.07) 0.086 0.666	0.94 (0.83–1.07) 0.249 0.348
Ethnicity							
Asian	8	3784/4540	0.95 (0.89–1.01) 0.248 0.125	0.89 (0.77–1.02) 0.258 0.102	0.98 (0.89–1.08) 0.585 0.682	0.96 (0.88–1.04) 0.396 0.322	0.90 (0.78–1.03) 0.401 0.111
Caucasian	2	537/489	1.18 (0.99–1.41) 0.082 0.062	1.38 (0.95–1.96) 0.105 0.095	1.28 (0.97–1.69) 0.201 0.082	1.30 (1.00–1.70) 0.115 0.047	1.18 (0.85–1.62) 0.236 0.322
Latin	1	51/37	0.94 (0.52–1.71) - 0.835	0.96 (0.23–3.93) - 0.955	0.37 (0.12–1.11) - 0.075	0.46 (0.16–1.34) - 0.157	1.97 (0.63–6.18) - 0.245
Cancer Type							
BCa	3	816/774	0.97 (0.83–1.12) 0.286 0.671	0.85 (0.61–1.17) 0.142 0.316	1.08 (0.87–1.33) 0.837 0.492	1.02 (0.84–1.25) 0.582 0.813	0.83 (0.61–1.12) 0.151 0.219
Other cancers	8	3556/4292	0.99 (0.89–1.11) 0.039 0.898	0.96 (0.83–1.11) 0.108 0.578	0.99 (0.89–1.09) 0.080 0.763	1.00 (0.86–1.16) 0.033 0.965	0.97 (0.84–1.11) 0.329 0.631
Source of control							
HB	5	1057/1231	1.02 (0.90–1.15) 0.297 0.746	0.99 (0.76–1.28) 0.123 0.939	1.08 (0.90–1.30) 0.193 0.415	1.06 (0.89–1.26) 0.341 0.539	0.98 (0.77–1.23) 0.082 0.837
PB	6	3315/3835	0.98 (0.87–1.10) 0.039 0.705	0.92 (0.79–1.08) 0.127 0.310	0.98 (0.88–1.08) 0.146 0.662	0.99 (0.85–1.14) 0.049 0.844	0.93 (0.80–1.08) 0.528 0.328
Lys460Thr							
Total	5	3019/3544	0.94 (0.67–1.32) 0.905 0.729	0.83 (0.36–1.93) 0.859 0.669	1.00 (0.66–1.50) 0.904 0.994	0.96 (0.67–1.40) 0.886 0.848	0.83 (0.36–1.93) 0.866 0.669
Ethnicity/source of control							
Asian/PB	5	3019/3544	0.94 (0.67–1.32) 0.905 0.729	0.83 (0.36–1.93) 0.859 0.669	1.00 (0.66–1.50) 0.904 0.994	0.96 (0.67–1.40) 0.886 0.848	0.83 (0.36–1.93) 0.866 0.669
Cancer Type							
BCa	1	375/375	0.76 (0.37–1.58) - 0.462	0.74 (0.17–3.35) - 0.700	0.77 (0.28–2.09) - 0.610	0.76 (0.33–1.76) - 0.526	0.75 (0.17–3.37) - 0.705
Other cancers	4	2644/3169	1.00 (0.68–1.46) 0.896 0.462	0.88 (0.32–2.42) - 0.799	1.05 (0.67–1.65) 0.868 0.823	1.02 (0.68–1.54) 0.886 0.919	0.87 (0.32–2.42) - 0.796
Lys87Glu							
Total	3	749/1919	1.05 (0.93–1.18) 0.968 0.430	1.11 (0.88–1.42) 0.953 0.377	0.96(0.78–1.18) 0.647 0.722	1.01 (0.83–1.22) 0.863 0.928	1.13 (0.93–1.38) 0.604 0.222
Ethnicity							
Caucasian	3	749/1919	1.05 (0.93–1.18) 0.968 0.430	1.11 (0.88–1.42) 0.953 0.377	0.96 (0.78–1.18) 0.647 0.722	1.01 (0.83–1.22) 0.863 0.928	1.13 (0.93–1.38) 0.604 0.222
Cancer Type							
BCa	2	543/1092	1.06 (0.91–1.22) 0.841 0.458	1.13 (0.85–1.51) 0.810 0.398	0.92 (0.72–1.17) 0.544 0.496	0.98 (0.78–1.23) 0.745 0.875	1.20 (0.94–1.53) 0.508 0.152
Other cancers	1	206/827	1.03 (0.83–1.28) - 0.756	1.07 (0.70–1.66) - 0.745	1.08 (0.74–1.59) - 0.684	1.08 (0.75–1.56) - 0.678	1.02 (0.72–1.43) - 0.921
Source of control							
HB	2	543/1092	1.06 (0.91–1.22) 0.841 0.458	1.13 (0.85–1.51) 0.810 0.398	0.92 (0.72–1.17) 0.544 0.496	0.98 (0.78–1.23) 0.745 0.875	1.20 (0.94–1.530 0.508 0.152
PB	1	206/827	1.03 (0.83–1.28) - 0.756	1.07 (0.70–1.66) - 0.745	1.08 (0.74–1.59) - 0.684	1.08 (0.75–1.56) - 0.678	1.02 (0.72–1.43) - 0.921

*P*_h_: *P* value of *Q*-test for heterogeneity test

*BCa* Bladder Cancer, *HB* hospital-based, *PB* population-based

### In silico analysis of MMP-8

**Fig. 4:**
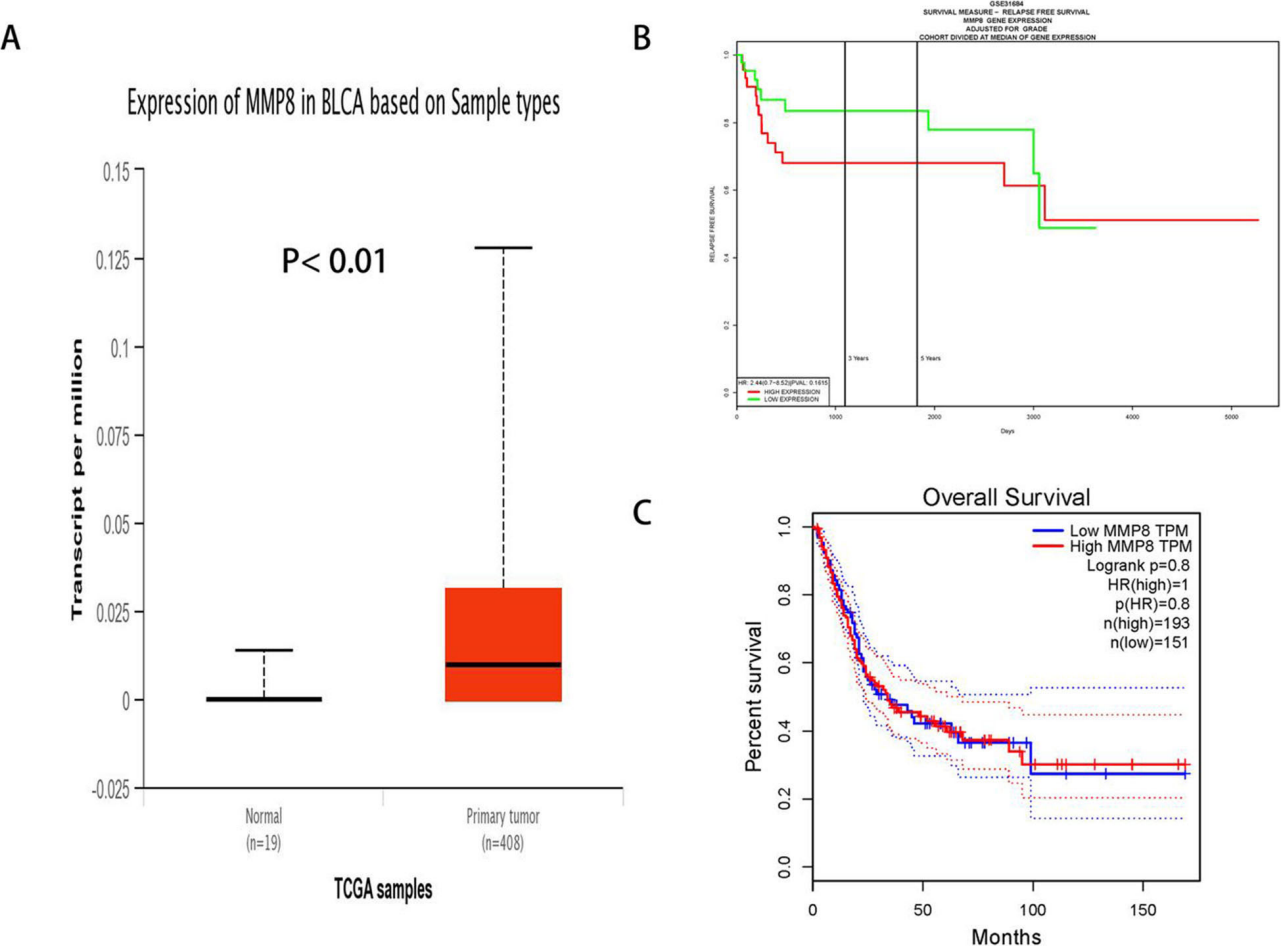
In silico analysis of *MMP-8* expression in bladder cancer (**a**). Association of the expression of *MMP-8* and the overall survival (OS) time among bladder cancer participants (**b**, **c**)

**Fig. 5:**
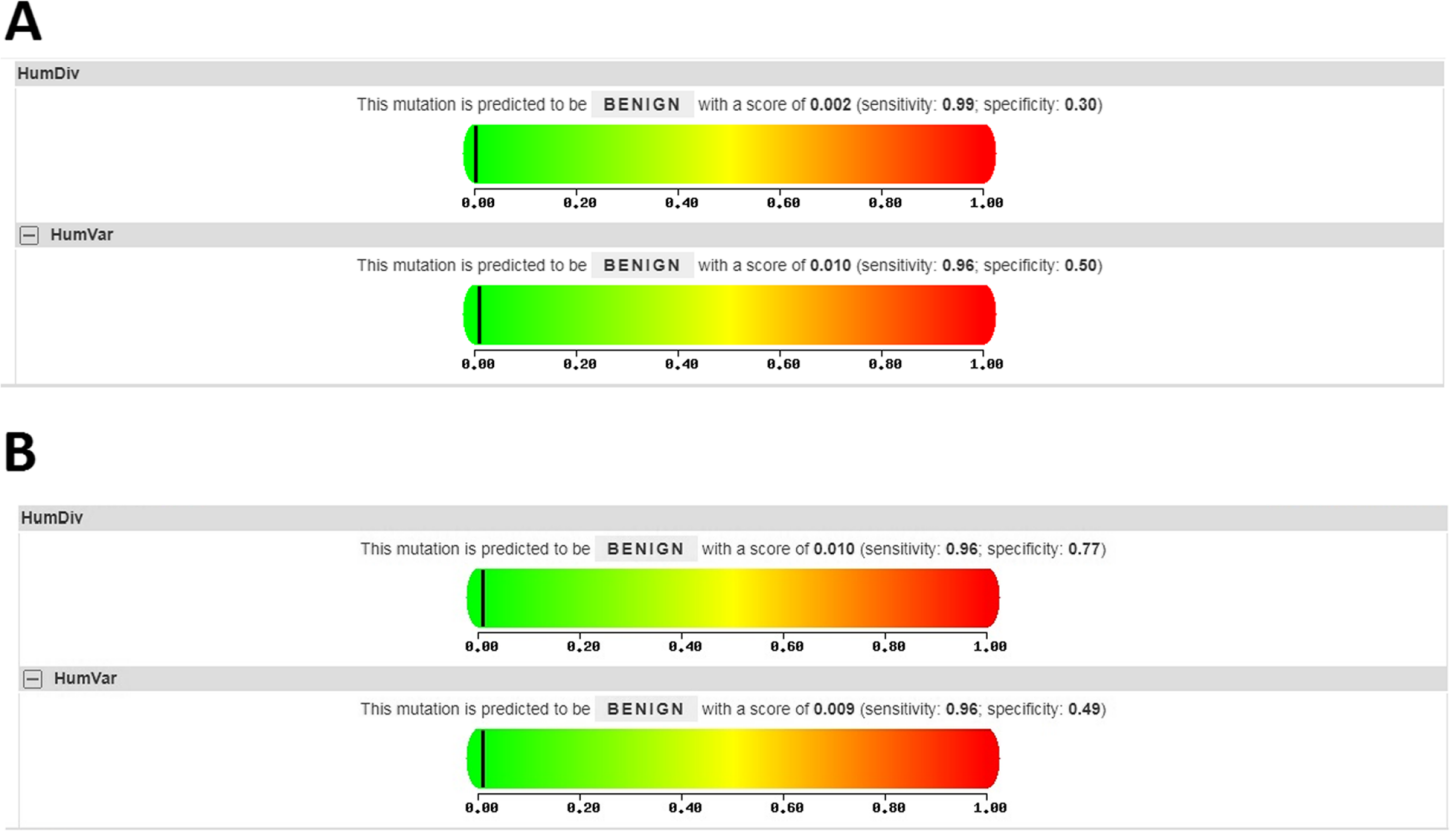
Polyphen2 bioinformatics tools for assessing the association between *MMP-8* Lys460Thr (K460 T, rs35866072, **a**), Lys87Glu (K87E, rs1940475, **b**) variants and protein damaging (The place of black line represents the score)

Results from the TCGA database, containing 408 primary tumor and 19 normal samples, revealed that *MMP-8* expression was elevated in bladder cancer tissue as compared to their control counterpart (*P* < 0.01, Fig. 4a). Furthermore, we investigated whether the *MMP-8* expression had an effect on the overall survival time of bladder carcinoma participants. However, neither higher *MMP-8* expression group nor lower expression group would have an impact on the patients’ overall survival time (*P* < 0.05, Fig. 4b, c). In addition, we adopted the Polyphen-2 bioinformatics tool to analyze the associations between *MMP-8* Lys460Thr (K460 T, rs35866072), and Lys87Glu (K87E, rs1940475) variants and protein damage. Mutations of these SNPs are predicted to be “BENIGN” with a score less than 0.05, which indicated that neither Lys460Thr nor Lys87Glu SNP may probably damage the protein of MMP-8 (Fig. 5).

### Publication bias

**Fig. 6:**
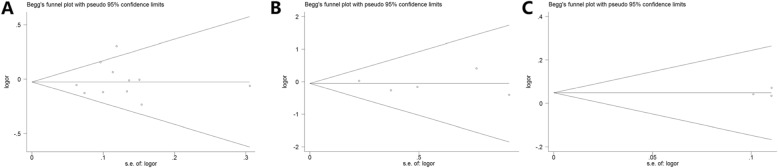
Begg’s funnel plot of standard error for investigating publication bias of *MMP-8* C-799 T (**a**), Lys460Thr (**b**), and Lys87Glu (**c**) variants under T-allele vs. C-allele genetic model

Both Egger’s and Begg’s funnel plot were employed for appraisal of the publication bias when evaluating *MMP-8* C-799 T, Lys460Thr, and Lys87Glu variants. No evidence of publication bias was acquired for *MMP-8* C-799 T polymorphism (T-allele vs. C-allele, t = 0.37, *P* = 0.722; TT versus CC, t = 0.26, *P* = 0.801; TC vs. CC, t = 0.59, *P* = 0.567; TT + TC versus CC, t = 0.62, *P* = 0.552; TT versus TC + CC, t = 0.07, *P* = 0.945). For *MMP-8* Lys460Thr variant: M-allele vs. W-allele, t = − 0.35, *P* = 0.752; MW vs. WW, t = − 0.71, *P* = 0.527; MM + MW vs. WW, t = − 0.68, *P* = 0.545. For Lys87Glu variant: A vs. G, t = 0.38, *P* = 0.771; AA vs. GG, t < − 0.01, *P* = 0.998; AG vs. GG, t = − 0.04, *P* = 0.975; AA + AG vs. GG, t = − 0.13, *P* = 0.916; AA vs. AG + GG, t = 1.71, *P* = 0.338. Outlines of the funnel plots were relatively symmetrical for overall cancer risk, implying no significant publication bias (Fig. 6).

## Discussion

The MMP-8 serves as one of the most efficient collagenases and plays an essential role in carcinoma invasion and metastasis. Previous research demonstrated that advanced metastatic stage and further poor prognosis of carcinoma might be related to elevated expression of MMPs [38, 39]. Additionally, it was hypothesized that the regulatory effect of MMPs could be associated with variations in the *MMP* genes. One group reported that the *MMP8* C-799 T variant might be related to breast carcinoma susceptibility and lymph node metastasis in Asians and Caucasians [21]. However, another group investigated *MMP8* variations among a representative Taiwanese breast carcinoma population and indicated no significant relationship between *MMP-8* C-799 T, and Lys460Thr polymorphisms and cancer risk [34]. Therefore, it is reasonable to summarize all eligible data and draw more accurate conclusions to evaluate the contribution of *MMP-8* polymorphisms to cancer risk. Furthermore, we employed the TCGA database and Polyphen2 bioinformatics tools to assess the role of *MMP-8* expression on cancer risk and survival time.

In the present study, a total of 8140 patients with malignant carcinoma and 10,529 control participants were investigated. For *MMP-8* C-799 T polymorphism, we observed no significant relationship with cancer risk (z-value = 0.79, *P*_heterogeneity_ = 0.068, *P* = 0.429, allelic contrast). Our finding was in agreement with the studies conducted by Hsiao et al., Huang et al., and Wieczorek et al [23, 31, 34]. In subgroup analysis by cancer type, this variation did not significantly confer susceptibility to urinary bladder cancer (z-value = 0.43, *P*_heterogeneity_ = 0.286, *P* = 0.671) and other cancers (z-value = 0.13, *P*_heterogeneity_ = 0.039, *P* = 0.898). In stratified analysis by race, a similar result was indicated in Asian (z-value = 1.54, *P*_heterogeneity_ = 0.248, *P* = 0.125) and Caucasian descendants (z-value = 1.87, *P*_heterogeneity_ = 0.082, *P* = 0.062). For *MMP-8* Lys460Thr variant, no positive correlation was found in the overall analysis (z-value = 0.35, *P*_heterogeneity_ = 0.905, *P* = 0.729). Similar results were indicated for Lys87Glu variant (z-value = 0.79, *P*_heterogeneity_ = 0.968, *P* = 0.430). Results from in silico analysis showed that *MMP-8* expression was elevated in bladder cancer tissue as compared to the control counterpart. However, both the higher and lower *MMP-8* expression groups did not have an impact on the patients’ overall survival time. Moreover, Polyphen-2 bioinformatics tool was also adopted to confirm the results of our present analysis. As the report for *MMP-8* C-799 T (rs11225395) variation was not available, the association between Lys460Thr (K460 T, rs35866072), and Lys87Glu (K87E, rs1940475) variants and protein damaging was further investigated. Mutations of Lys460Thr and Lys87Glu were predicted to be “BENIGN” with a score less than 0.05, which indicated that these SNPs do not damage MMP-8 protein, and are in agreement with the conclusions of the current analyses.

In addition, several limitations of the present study should be clarified. First of all, the number of enrolled studies for subgroup analysis remains insufficient, which exhibits fairly limited statistical power. Only five studies for *MMP-8* Lys460Thr SNPs and three for Lys87Glu polymorphism were acquired based on the selection criteria. As regard to C-799 T variant, only two case-control studies were focused on Caucasian population and one was based on Latin descendants. In addition, tumor stage and grade may potentially influence the results of the present analysis. We tried to further evaluate this effect in more details; however, raw data of eligible studies remains insufficient. More efficient investigations are still required to further strengthen the statistical power. Last but not least, *P* value for HWE was less than 0.05 in five of the included articles [30, 31, 33–35], which might be exposed to unknown bias factors.

Despite these limitations, some key advantages should be acknowledged. First, all eligible case-control studies according to the selection criteria were obtained and the statistical efficiency was enhanced remarkably. Second, no obvious publication bias was indicated by Egger’s and Begg’s funnel plot, which showed that the findings of the current analysis can be considered reliable. Additionally, NOS scores of the enrolled studies were more than 6, which indicated a high methodological quality of each article.

## Conclusions

Taken together, based on the currently published data, our study showed evidence that *MMP-8* C-799 T, Lys460Thr, and Lys87Glu variants are not participant with the susceptibility of cancer. Further well-designed investigations are still warranted to confirm this conclusion in more detail.

## Data Availability

All the data generated in the present research is contained in this manuscript.

## Abbreviations

BCa: Bladder cancer
HB: Hospital-based
HCa: Hepatocellular carcinoma
HWE: Hardy-Weinberg equilibrium of controls
M-allele: Mutant allele
MM: Malignant melanoma
NA: Not available
NOS: Newcastle–Ottawa Scale
PB: Population-based
PCR-RFLP: Polymerase chain reaction and restrictive fragment length polymorphism
RT: Real time
W-allele: Wild-type allele
